# Asymptomatic Giant Lipoma of the Right Ventricular Outflow Tract: A Case Report

**Published:** 2017-07

**Authors:** Fariba Bayat, Zargham Hossein Ahmadi, Neda Behzadnia, Atosa Dorudinia, Alireza Jahangirifard

**Affiliations:** 1 *Cardiovascular Research Center, Shahid Beheshti University of Medical Sciences, Tehran, Iran.*; 2 *Lung Transplantation Research Center, National Research Institute of Tuberculosis and Lung Diseases (NRITLD), Shahid Beheshti University of Medical Sciences, Tehran, Iran.*

**Keywords:** *Ventricular outflow tract obstruction*, *Heat neoplasms*, *Lipoma*

## Abstract

Cardiac lipomas are extremely rare tumors of the heart. They are usually symptomatic and rarely may be found incidentally in autopsies. Here we describe a 23-year-old healthy man, in whose physical examination for employment a murmur was found incidentally. Transthoracic and then transesophageal echocardiographic examination showed a 4-cm oval-shaped mass in the right ventricular outflow tract. He underwent elective surgery, during which the tumor was removed under cardiopulmonary bypass and aortic-cross clamping via right atriotomy. The postoperative course was uneventful, and the patient was in good condition at 1 year’s follow-up.

## Introduction

Heart tumors are rare, and about 75% of primary heart tumors are benign. Myxomas are encountered as the most benign tumors. Lipomas are very rare, but most of them cause obstructive symptoms such as dyspnea and less frequently arrhythmia.^1^ Herein, we present a case with a giant lipoma of the right ventricular outflow tract. 

## Case Report

A 23-year-old healthy man underwent a routine physical examination as a prerequisite for employment in a company. The examination revealed a grade IV/VI systolic murmur at the left sternal border with no radiation to any other area. Thrill was also palpated on the left sternal border.

**Figure 1:**
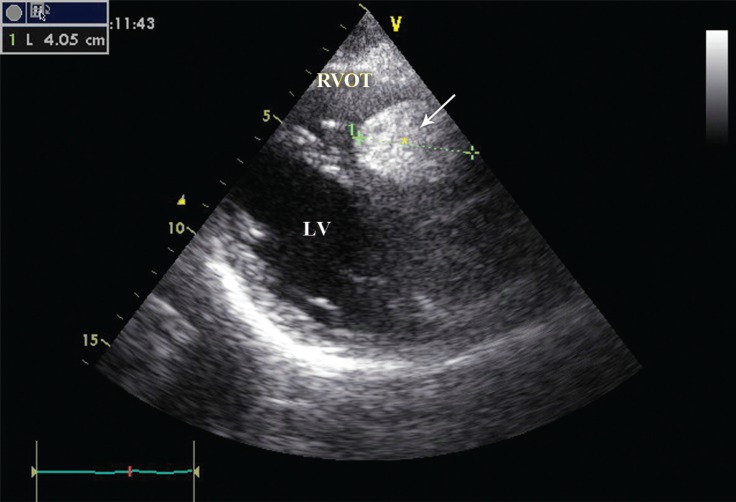
Two-dimensional parasternal long-axis view in transthoracic echocardiography shows a large mass in the right ventricular outflow tract (arrow).

LV, Left ventricle; RVOT, Right ventricular outflow tract

Transesophageal (TEE) illustrated a large (4 cm), oval-shaped, hypoechoic homogenous mass with smooth borders attached to the right ventricular outflow tract (RVOT) wall adjacent to the pulmonic valve, compatible with a benign tumoral mass (Figure 1). Electrocardiography (ECG), chest X-ray, and all lab data were normal. No other workups were done.

**Figure 2:**
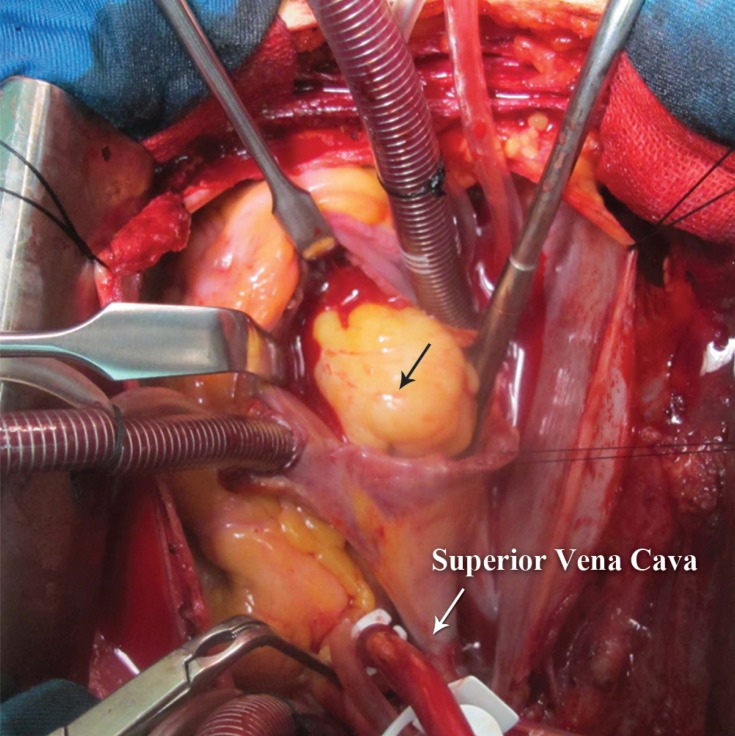
Protrusion of the mass (black arrow) in the right ventricular outflow tract through the tricuspid valve immediately after right atriotomy.

**Figure 3:**
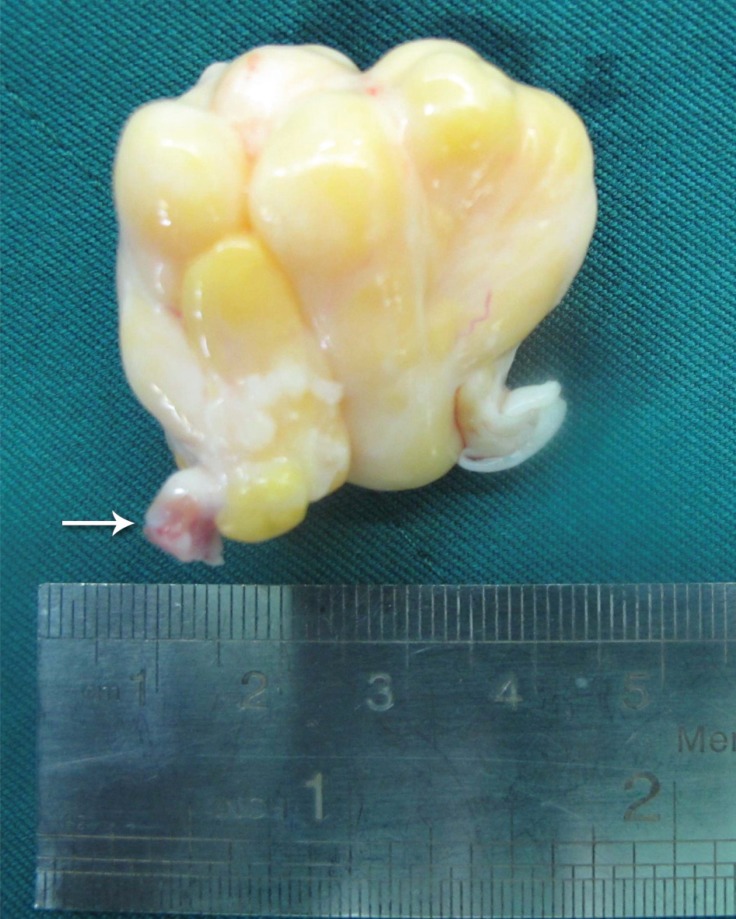
Yellow, lobulated mass in the right ventricular outflow tract after complete excision. The narrow stalk of the mass is shown with an arrow.

The patient underwent resection of the mass. Bicaval cannulation was done, cardiopulmonary bypass was applied, and cardiac arrest was obtained using a cold blood cardioplegia solution without the induction of hypothermia. The mass was approached via right atriotomy. A large (4 × 4 cm), yellow, lobulated, well-encapsulated mass was seen. The mass was attached to the lateral wall of the RVOT and the anterior cusp of the tricuspid valve (Figure 2 and Figure 3). The mass was excised and there was no need to repair the tricuspid valve. The postoperative course was uneventful.

**Figure 4:**
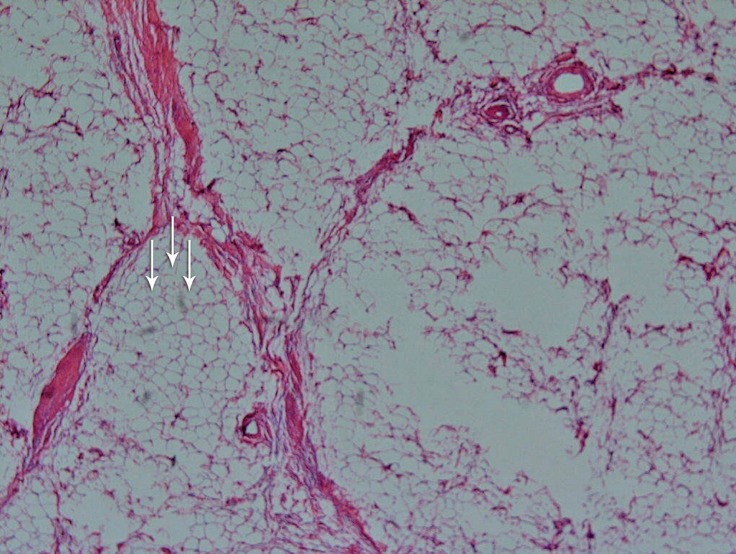
Microscopic evaluation of the tumor in the right ventricular outflow tract. The lipoma (shown with multiple arrows) consists of mature fat cells, with only a slight variation in cellular size and shape (hematoxylin and eosin staining, 100 X).

Microscopically, the mass was composed of mature fat cells, varying slightly in size and shape. The nuclei were fairly uniform, and there was an absence of nuclear hyperchromasia (Figure 4).

The patient was discharged on the 6th postoperative day, and he was doing well at 1 year’s follow-up.

## Discussion

Benign non-myxoma tumors of the heart account for 2% to 10% of all cardiac tumors. Most of these tumors are asymptomatic and are found incidentally in computed tomography (CT) or magnetic resonance imaging (MRI).^1^^, ^^2^ Lipomas in the right ventricle (RV) are extremely rare.

The clinical manifestation of these tumors depends on their location. Tumors in the left ventricle may present with syncopal attacks,^3^ those in the aorta may cause sudden death,^4^ and the ones in the right atrium^5^ or in the epicardium^6^ may be asymptomatic. Moreover, this group of tumors may also present with cardiomegaly.^7^

Tumors that are adjacent to the valves may present with murmurs or symptoms of valvular obstruction, while those located in the right atrium, RV, or interatrial septum are more prone to arrhythmias.^8^

In our patient, the tumor, which seemed large, was in close proximity of the pulmonary valve, inducing a high-grade murmur. Nevertheless, from a functional point of view, it was not obstructive to the pulmonary artery, which explains the absence of RV wall hypertrophy.

Non-myxomatous benign tumors are seen in lower age than myxomatous tumors.^2^ Despite the reports on patients in old ages,^5^ the young age of our patient and also the location of the tumor was suggestive of a benign non-myxomatous mass. Nevertheless, what should be borne in mind is that in 5% of cases, myxomas may be seen in the RV.^2^

We did not perform any additional workups because according to TEE and lab data, there was no evidence of involvement in other places and surgery was indicated. The operation was done as routine for all heart tumors. Still, as incisions over the RVOT may induce arrhythmias,^9^ we opted to approach the tumor via the right atrium and perform right ventriculotomy only if there was any difficulty in approaching the mass. Fortunately, the whole operation was performed easily through the right atrium. It is worthy of note that no finding on CT scan or MRI would have changed our decision for surgery.

## Conclusion 

Large heart tumors, even in the RVOT, may be asymptomatic. Evens so, a meticulous physical examination and a precise auscultation of the heart may confer a clue for the diagnosis. Surgical excision is necessary. If chest radiograph and echocardiography fail to show any evidence of malignancy, CT scan or MRI may not be necessary. 

## Notes:

***This paper should be cited as: ***Bayat F, Ahmadi ZH, Behzadnia N, Dorudinia A, Jahangirifard A. Asymptomatic Giant Lipoma of the Right Ventricular Outflow Tract: A Case Report. J Teh Univ Heart Ctr 2017;12(3):142-144.
